# Time-Resolved Study of Nanomorphology and Nanomechanic Change of Early-Stage Mineralized Electrospun Poly(lactic acid) Fiber by Scanning Electron Microscopy, Raman Spectroscopy and Atomic Force Microscopy

**DOI:** 10.3390/nano7080223

**Published:** 2017-08-17

**Authors:** Mengmeng Wang, Yin Cai, Bo Zhao, Peizhi Zhu

**Affiliations:** 1 School of Chemistry and Chemical Engineering, Yangzhou University, Yangzhou 225002, China; meslierw@hotmail.com (M.W.); caiyin525@hotmail.com (Y.C.); 2 Jiangsu Collaborative Innovation Center of Biomedical Functional Materials and Jiangsu Key Laboratory of Biofunctional Materials, School of Chemistry and Materials Science, Nanjing Normal University, Nanjing 210023, China; zhaobo@njnu.edu.cn

**Keywords:** poly(lactic acid), electrospun, atomic force microscopy (AFM), scanning electron microscope (SEM), nanomechanical properties, biomineralization

## Abstract

In this study, scanning electron microscopy (SEM), Raman spectroscopy and high-resolution atomic force microscopy (AFM) were used to reveal the early-stage change of nanomorphology and nanomechanical properties of poly(lactic acid) (PLA) fibers in a time-resolved manner during the mineralization process. Electrospun PLA nanofibers were soaked in simulated body fluid (SBF) for different periods of time (0, 1, 3, 5, 7 and 21 days) at 10 °C, much lower than the conventional 37 °C, to simulate the slow biomineralization process. Time-resolved Raman spectroscopy analysis can confirm that apatites were deposited on PLA nanofibers after 21 days of mineralization. However, there is no significant signal change among several Raman spectra before 21 days. SEM images can reveal the mineral deposit on PLA nanofibers during the process of mineralization. In this work, for the first time, time-resolved AFM was used to monitor early-stage nanomorphology and nanomechanical changes of PLA nanofibers. The Surface Roughness and Young’s Modulus of the PLA nanofiber quantitatively increased with the time of mineralization. The electrospun PLA nanofibers with delicate porous structure could mimic the extracellular matrix (ECM) and serve as a model to study the early-stage mineralization. Tested by the mode of PLA nanofibers, we demonstrated that AFM technique could be developed as a potential diagnostic tool to monitor the early onset of pathologic mineralization of soft tissues.

## 1. Introduction

Poly(lactic acid) (PLA)-based biomaterials as biodegradable and biocompatible materials are widely used in bone tissue engineering [1,2]. Over recent years, there are several established techniques to prepare a variety of polymeric fibers from nano to micro scale, including centrifugal spinning [3], electrospinning technique and gyratory methods [4]. The most appealing electrospinning characteristic has been shown to be the mimicking nano-scale fibrous topography of extracellular matrix (ECM) in the tissue engineering field [5,6,7]. This technique allows for the production of polymer fibers with diameters varying from 3 nm to greater than 5 μm, which are capable of supporting a wide variety of cell types [8,9]. Nanofibers produced by electrostatic spinning have been successfully used as scaffold materials for tissue engineering due to their large surface-area-to-volume ratio, high porosity, and delicate microstructure [10,11,12]. The electrospinning process has shown great potential in various applications due to its ability of facile producing high surface-to-volume fibrous structure [13,14,15].

Biomineralization is the complex process by which living organisms form minerals and biomineralization processes often lead to the hardening or stiffening of the existing tissues such as mineralized skeletons [16]. Inspired by natural biomineralization processes, some researchers deposited minerals on the surface of a class of materials including proteins [17], inorganic-organic composites, and synthetic polymers [18,19,20]. The organic matrix is used as a template to control the formation of inorganic matter for preparing material with microstructure characteristic and biological function. Apatite layers formed on the surface of PLA can improve the hydrophilicity of PLA materials and enhance the cell adhesion on PLA surface [21,22,23,24,25], which has a better cell response and stimulation of bone regeneration rate [26]. VEGF (Vascular endothelial growth factor) loading onto mineralized PLA gained better regenerative ability of damaged bone tissue by stimulating vascularization and tissue perfusion [27]. 

Although mineralization of hydroxyapatite in electrospun nanofibrous Poly(lactic acid) scaffolds has been well studied [28], the detailed nanomechanical and surface properties change of PLA during early-stage mineralization process has not yet become clear. Typically, the mineralization process was initiated by immersing the electrospun scaffold in the simulated body fluids (SBF) at 37 °C for varying periods of time. Due to the high temperature of SBF, minerals more quickly develop on the surface of PLA fibers after incubation in SBF and usually become well mineralized after 3~4 days. In this paper, electrospun PLA nanofibers were soaked in SBF for different periods of time at low temperature to simulate the slow biomineralization process and scanning electron microscopy was used to observe the surface morphology and mineral deposition of electrospun PLA fibers. In addition, time-resolved Raman spectroscopy was used to characterize the chemical nature of formed minerals on the surface of PLA fiber. Atomic force microscope (AFM) is a useful tool for obtaining insights into the mechanism of nanofibers-minerals interactions. Therefore, AFM were also used to observe the process of PLA fiber mineralization and to analyze the nanomechanical change of PLA nanofibers. The final goal of the study is to explore the possible application of mineralized PLA nanofibers in the field of bone tissue engineering. Biomineralization of PLA nanofibers was further investigated to verify whether it could be a suitable matrix for bone cell growth. Therefore, the next step of our work will be evaluating the biocompatibility of mineralized PLA nanofibers by using several types of bone cells to provide preliminary evidence for future medical applications.

## 2. Materials and Methods

### 2.1. Chemicals and Materials

Sodium chloride (NaCl), sodium bicarbonate (NaHCO_3_), potassium chloride (KCl), Trihydrate potassium hydrogen phosphate (K_2_HPO_4_·3H_2_O), magnesium chloride hexahydrate (MgCl_2_·6H_2_O), calcium chloride (CaCl_2_), sodium sulfate (Na_2_SO_4_), Dichloromethane (DCM) and hydrochloric acid (HCl) were purchased from Sinopharm Chemical Reagent Co., Ltd. (Shanghai, China), Tris(hydroxymethyl)aminomethane (HOCH_2_)_3_CNH_2_ were purchased from Sigma-Aldrich (VETEC, Milwaukee, WI, USA), poly(lactic acid) (PLA) were purchased from NatureWorks (Blair, NE, USA). All the chemicals were of analytical reagent grade. All of the solutions were freshly prepared using deionized double-distilled water.

### 2.2. Preparation of Electrospinning Solutions

PLA nanofibers were produced by way of electrospinning as previously reported. Slightly, PLA (0.75 g) was dissolved in DCM (5 mL) to get a viscous solution (0.15 g/mL) for electrospinning for 12 h at room temperature.

### 2.3. Electrospinning of PLA Nanofibers 

Electrospinning solution (about 5 mL) was drawn into 15 mL plastic syringes connected by a plastic tube with a metal capillary tube. The syringe was maintained at a flow rate of 1 mL/h. Electrospinning was carried out at room temperature of 20 °C and at environmental humidity of 30%. In electrospinning, the nozzles on its axis at a linear speed of 100 mm/min, and the lateral distance of 150 mm to keep moving. Fibers were collected in aluminum foil surface and drying process was conducted at the condition of 40 °C for 24 h.

### 2.4. Preparation of Simulated Body Fluid (SBF) and Mineralization in SBF

The SBF was prepared according to literature [29,30] to mimic the concentration of human plasma. PLA fiber film was cut into square pieces with dimension of 5 mm × 5 mm, soaked in SBF for different time periods (0, 1, 3, 5, 7 and 21 days) at 10 °C. At each time point, samples were removed, gently washed with ultrapure water, and then dried in air for characterization. Sample names were labeled a, b, c, d, e and f, corresponding to the number of soaking time are 0, 1, 3, 5, 7 and 21 days.

### 2.5. Characterization of Mineralized PLA Nanofibers

#### 2.5.1. Scanning Electron Microscopy (SEM) Analysis of Mineralized PLA Nanofibers

For morphological observation, the mineralized PLA fiber pieces were observed using SEM (S-4800II field emission scanning electron microscopy, Hitachi, Tokyo, Japan) at an accelerating voltage of 15 kV. 

#### 2.5.2. Raman Analysis of Mineralized PLA Nanofibers

Chemical structures of the products were detected by Raman (DXR, GX-PT-2412, Thermo, New York, NY, USA). An excitation wavelength of 780 nm and a spectral resolution of 1 cm^−1^ were used in this work. The spectra were recorded in the range of 400–3200 cm^−1^.

#### 2.5.3. Atomic Force Microscopy (AFM) Imaging and Data Analysis

An Icon AFM (Dimension Icon, Bruker, Santa Barbara, CA, USA) was used to image the mineralized samples. For imaging mineralized samples, we used a 500 nN force, a scanning rate of 1 Hz and RTEAPA (MPP-21110-10) cantilevers with spring constant of 3 Nm^−1^. Tip radius was calibrated against a polystyrene standard provided by Bruker. The measured value of the tip radius was 10 nm. Both samples imaging was carried out in Scan AsystPeak Force QNM (Quantitative Nanomechanical Mapping) mode. Force-distance curves were collected by nanoindentation of the sample in a point-by-point fashion. The maximum force (peak force) was controlled at each pixel to obtain force-distance curves, which are then used as feedback signal. Image processing and nanomechanical properties analysis was performed with the NanoScope Analysis software (Bruker, Santa Barbara, CA, USA). 3D topography images were generated from NanoScope Analysis software. Surface Roughness measurements were carried out using NanoScope Analysis software too.

## 3. Results and Discussion

### Characterization of Mineralized PLA Nanofibers

**Figure 1:**
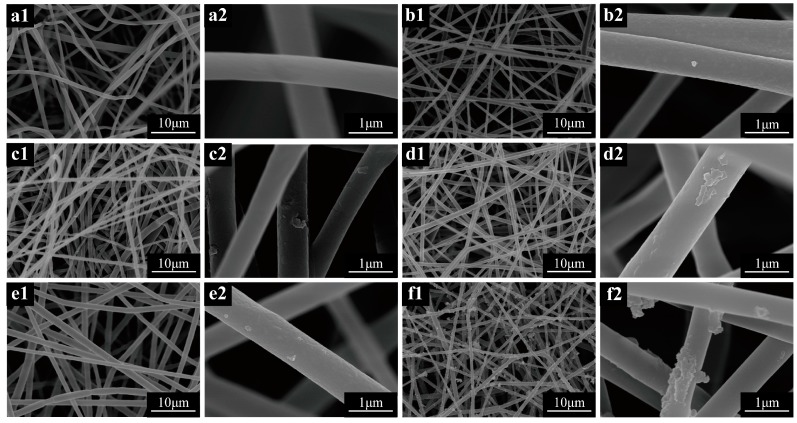
SEM photographs of PLA nanofibers immersed in SBF for different time: (**a1**,**a2**) 0-day; (**b1**,**b2**) 1-day; (**c1**,**c2**) 3-day; (**d1**,**d2**) 5-day; (**e1**,**e2**) 7-day; (**f1**,**f2**) 21-day.

After being immersed in SBF for 0–21 days, the morphology of the PLA nanofibers was observed by SEM. Minerals were found to be gradually deposited onto PLA nanofibers as time going on. As shown in Figure 1, minerals began to form and distribute evenly on the surface of poly(lactic acid) nanofibers from the first day (Figure 1(a1,a2)), and the amount of minerals increased continuously as the immersion time prolonged. Finally, PLA nanofibers were enwrapped with mineral layer after 21 days of mineralization (Figure 1(f1,f2)). In addition, differences in fiber morphology were clearly detected by SEM as the immersion time increased. The surface of the 0 day fiber sample was substantially smooth (Figure 1(a1,a2)), but the surface of the samples after 7 days and 14 days of immersion time show some subtle structures such as plate-like (Figure 1(c1,c2)) or flaky features (Figure 1(d1,d2)) [31]. Particularly, the surface of PLA fiber sample after 21 days’ immersion was found to be much rougher (Figure 1(f1,f2)). 

**Figure 2:**
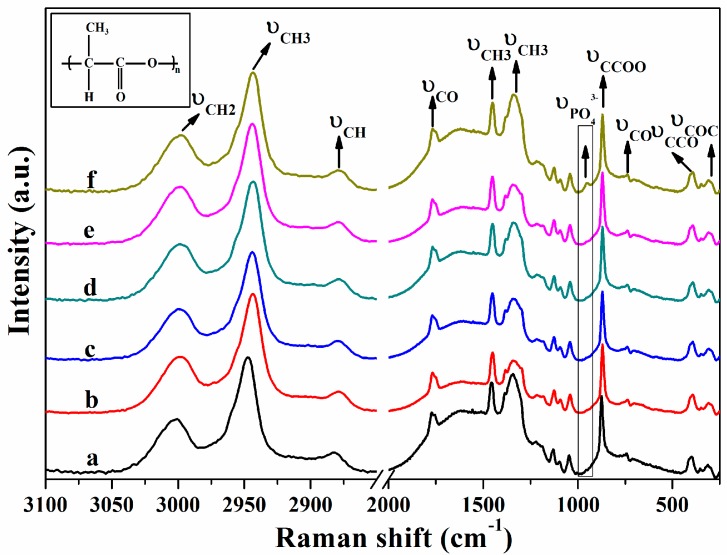
Raman spectra of PLA in the range of 250–2000 cm^−1^ and 2850–3100 cm^−1^ by being soaked in SBF for different time: (**a**) 0-day; (**b**) 1-day; (**c**) 3-day; (**d**) 5-day; (**e**) 7-day; (**f**) 21-day.

The Raman spectra of PLA nanofibers soaked in SBF for different periods of time were shown in Figure 2. Absorption bands between 3100 cm^−1^ and 2800 cm^−1^ were attributed to C–H stretching modes of CH and CH_3_ groups. The CH_3_ stretching modes appeared at 3014 cm^−1^ and 2960 cm^−1^. The CH stretching mode was observed at 2877 cm^−1^, which is consistent with the previous literature [32]. The CH_3_ symmetric and asymmetric deformation modes were observed at 1382 cm^−1^ and 1453 cm^−1^. The typical stretching of C=O group was centered at 1769 cm^−1^. Besides, bands at 1085 cm^−1^, 873 cm^−1^, 739 cm^−1^, 398 cm^−1^ and 308 cm^−1^ can be assigned to the COC, C–COO stretching modes, C=O, CCO, and COC deformation vibration of PLA [33,34,35]. The P–O stretching in PO_4_^3−^ groups at 960 cm^−1^ appeared after PLA fiber sample was immersed in SBF for 21 days, indicating that the apatite minerals were deposited on the surface of PLA nanofibers [28,36]. 

**Figure 3:**
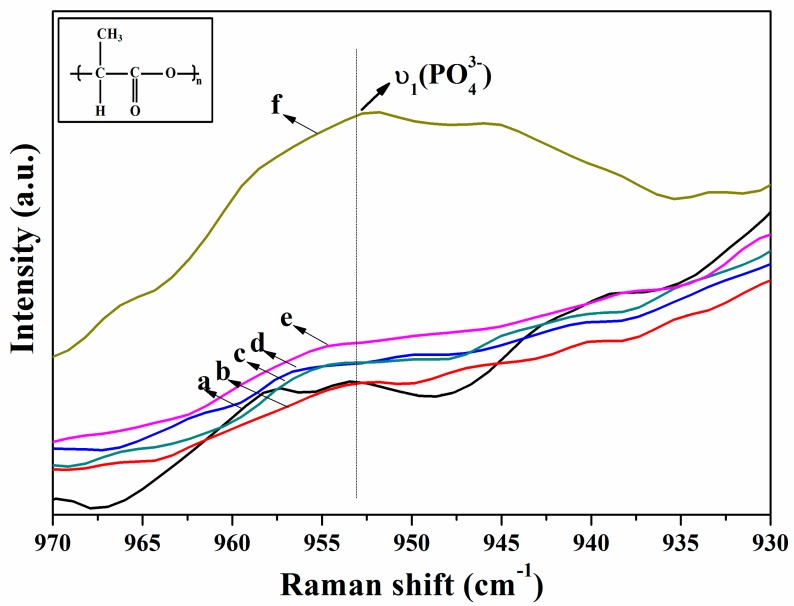
Raman spectra of PLA in the range of 930–970 cm^−1^ by being soaked in SBF for different time: (**a**) 0-day; (**b**) 1-day; (**c**) 3-day; (**d**) 5-day; (**e**) 7-day; (**f**) 21-day.

As shown in Figure 3, the apatite band at 960 cm^−1^ gradually increased with the mineralization time, indicating the amount of deposited apatite on the surface of PLA fiber was increasing. Time resolved Raman spectroscopy analysis could confirm that apatites were deposited on the PLA nanofibers after 21 days of mineralization. However, there is no apparent signal change among several Raman spectra before 21 days as shown by Figure 2 since the amount of mineral deposit on the surface of PLA nanofibers during early-stage of mineralization is small.

**Figure 4:**
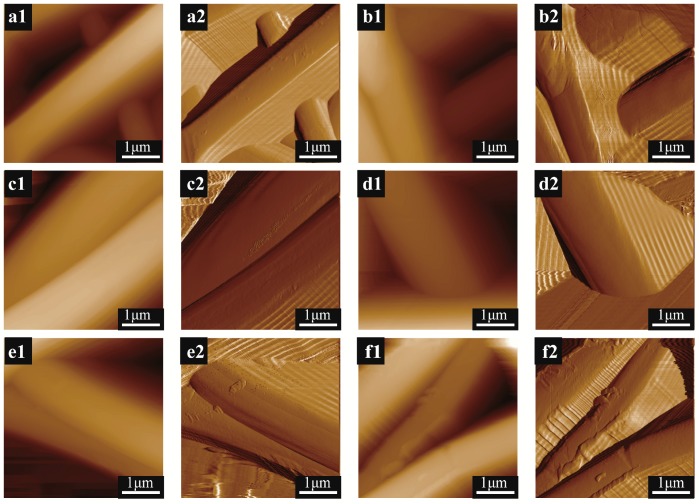
Height (**a1**,**b1**,**c1**,**d1**,**e1**,**f1**) and Peak Force Error (**a2**,**b2**,**c2**,**d2**,**e2**,**f2**) images of PLA nanofibers with different mineralization time: (**a1**,**a2**) 0-day; (**b1**,**b2**) 1-day; (**c1**,**c2**) 3-day; (**d1**,**d2**) 5-day; (**e1**,**e2**) 7-day; (**f1**,**f2**) 21-day.

**Figure 5:**
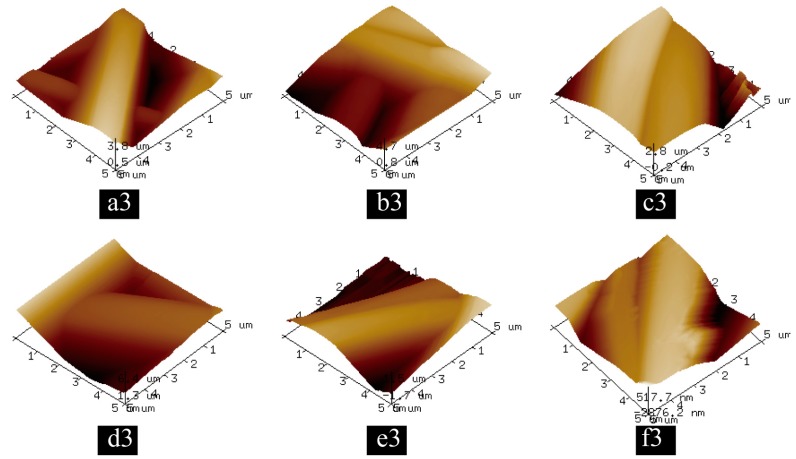
3D topography AFM images of PLA nanofibers along with different mineralization time: (**a3**) 0-day; (**b3**) 1-day; (**c3**) 3-day; (**d3**) 5-day; (**e3**) 7-day; (**f3**) 21-day.

Surface morphology of PLA nanofibers was observed by high-resolution atomic force microscopy. As shown in Figure 4a–f, sheet-shaped minerals appeared on the surface of the PLA samples after immersion in SBF for 0, 1, 3, 5, 7 and 21 days. Figure 4(b1,b2,e1,e2) showed a clear tendency for the deposited minerals to grow and become thicker and more uniform with increasing immersion time [37]. As observed in Figure 4(f1,f2), minerals were homogenously dispersed on the surface of the nanofibers [38]. As shown in Figure 5(a3,b3), the minerals could not be clearly observed, while in Figure 5(f3), the dispersed minerals were obviously observed on the surface of the nanofibers, 3D mapping (Figure 5(a3–f3)) further demonstrated the increasing number of mineralized material adhering to the surface of the PLA nanofibers along with the increasing immersion time. 

Analysis of the force-curves was performed by the Peak Force Tapping technology and the topography and Young’s Modulus were measured by ScanAsyst^®^ (Bruker, Santa Barbara, CA, USA). To obtain the Young’s Modulus, the retrace curve was fitted by the software using the Derjaguin-Muller-Toporov (DMT) Model [39]:

(1) $${F-F}_{adh}=\frac{4}{3}E^{*}\sqrt{R{\left({d-d}_{0}\right)}^{3}}$$

where *F* − *F_adh_* was the force on the cantilever (*F*) relative to the adhesion force (*F_adh_*), *R* was the tip end radius, and *d* − *d*_0_ was the deformation of the sample.

**Figure 6:**
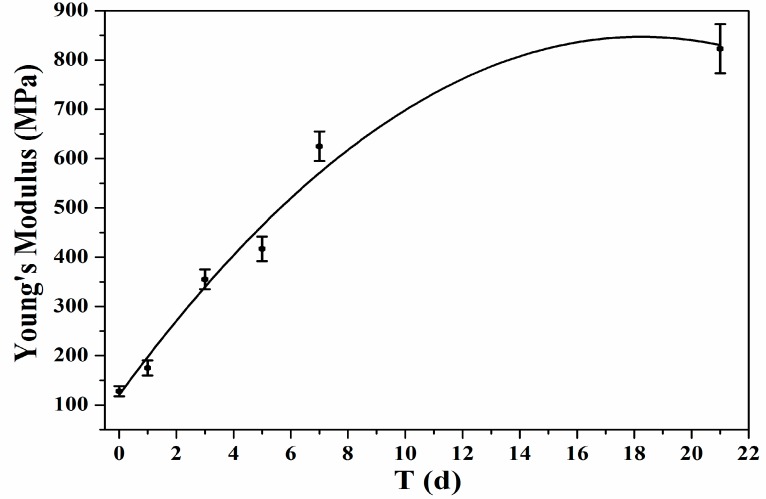
Young’s Modulus figure of PLA nanofibers with different mineralization times.

As shown in Table 1 and by Figure 6, the measured values of Young’s Modulus of PLA fiber increased with the increasing mineralization time. Young’s Modulus of PLA fiber was 128 ± 5 MPa. After 3 days’ immersion in SBF, Young’s Modulus of PLA fiber almost tripled to 355 ± 13 MPa. After 21 days’ immersion in SBF, Young’s Modulus of PLA fiber reached 823 ± 30 MPa, indicating the apatite layer on the surface of PLA nanofibers significantly changed the nanomechanic properties of PLA fibers.

**Figure 7:**
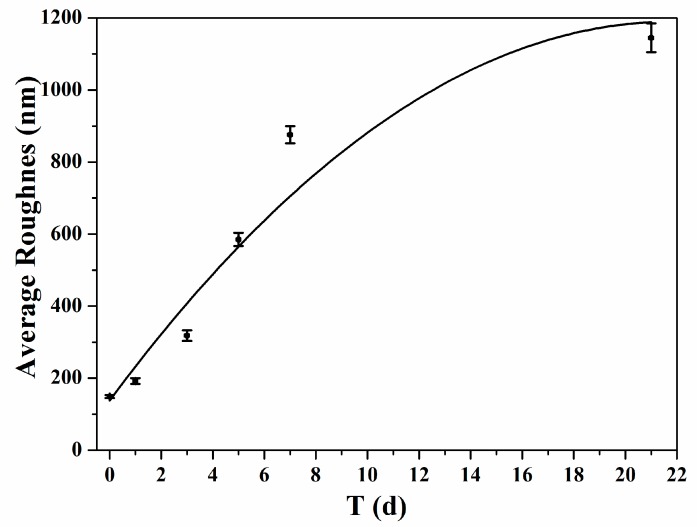
Average Roughness figure of PLA nano fibers with different mineralization time.

Roughness data (Table 1) were obtained from different regions on each sample. Figure 7 showed the surface roughness of PLA fibers immersed SBF for different periods of time. As seen in Table 1, roughness analysis showed average roughness of untreated PLA is 149 ± 4 nm. After 1 day of mineralization, the roughness of PLA has not changed significantly (192 ± 8 nm). The roughness of PLA fibers almost tripled to 585 ± 18 nm after 5 days’ immersion in SBF. As the immersion time in SBF went on, the roughness kept increasing and reached to 1145 ± 40 nm after been treated in SBF for 21 day [40,41]. The almost eight-fold increase in roughness was caused by deposit of mineral particles on the surface of PLA fibers. Both height and topographic images indicate the relative homogeneity of PLA before immersed in SBF (Figure 5(a1) and Figure 6(a3)). The smoother morphology of the mineral-covered PLA surface was caused by a thin and even full coverage of mineral deposit, whereas the coarser morphology of PLA fibers after mineralized for long time was caused by a thicker and irregular mineral coverage. As shown by Table 1, measured surface area of PLA fibers are increased with the mineralization time, which are consistent with modulus data of PLA fibers [42]. The increased surface roughness indicated more mineral has been deposit on the surface of PLA. 

Some pathologic calcification including artery calcification, cardiac valve calcification, and myositis ossificans are associated with the undesired biomineralization [43,44]. The mechanisms of coronary heart disease caused by arteriosclerosis and then straitness could also be involved with unwanted mineral deposition along collagen fibers and associated nanomechanic changes of tissue. It is, therefore, of great importance to detect the early-stage change accompanying with the formation of undesirable biominerals in organisms although some detailed mechanism of biomineralization remain debated [45,46,47]. These electrospun PLA nanofibers with delicate structure could be a useful model for studying apatite formation and observing the nanomorphology change and investigating nanomechanical properties during early biomineralization process. The time-resolved nanomechanic data and high-resolution images provided by AFM may potentially serve as a spectroscopic tool to diagnose the early onset of pathologic mineralization of soft tissues in situ.

## 4. Conclusions

An early-stage mineralization study from electrospun nanofibrous PLA scaffolds was carried out in the SBF. In the study, high-resolution SEM, Raman spectroscopy and AFM were used to reveal nanostructural changes to mineralized PLA nanofibers. The results indicated that the deposited minerals were apatite with plate-like or flaky-like structures. The Surface Roughness, Young’s Modulus and Surface Area of the PLA nanofibers significantly increased with the time of mineralization, which could be used to monitor early-stage nanomorphology and nanomechanical changes of PLA nanofibers. Using electrospun PLA nanofibers as a model to study early-stage mineralization, we demonstrated that AFM techniques could be developed as a potential diagnostic tool to monitor the early onset of the pathologic mineralization of tissues.

## Figures and Tables

**Table 1:** Young’s Modulus, Average Roughness and Surface Area results of PLA nanofibers with different mineralization time.

Physical Matrix	Mineralization Days	Young’s Modulus (MPa)	Average Roughness (nm)	Surface Area (μm^2^)
Mineralized Condition	SBF (0-day)	128 ± 5	149 ± 4	1.04 ± 0.14
	SBF (1-day)	175 ± 8	192 ± 8	1.21 ± 0.17
	SBF (3-day)	355 ± 13	318 ± 15	1.99 ± 0.21
	SBF (5-day)	417 ± 18	585 ± 18	2.01 ± 0.23
	SBF (7-day)	625 ± 21	876 ± 24	2.14 ± 0.25
	SBF (21-day)	823 ± 30	1145 ± 40	2.28 ± 0.27
